# Combination of the amide‐to‐triazole substitution strategy with alternative structural modifications for the metabolic stabilization of tumor‐targeting, radiolabeled peptides

**DOI:** 10.1002/psc.3654

**Published:** 2024-09-11

**Authors:** Xabier Guarrochena, Maximilian Anderla, Philipp Salomon, Irene V. J. Feiner, Berthold A. Nock, Theodosia Maina, Thomas L. Mindt

**Affiliations:** ^1^ Institute of Inorganic Chemistry, Faculty of Chemistry University of Vienna Vienna Austria; ^2^ Vienna Doctoral School in Chemistry University of Vienna Vienna Austria; ^3^ Department of Biomedical Imaging and Image Guided Therapy, Division of Nuclear Medicine Medical University of Vienna Vienna Austria; ^4^ Joint Applied Medicinal Radiochemistry Facility University of Vienna and Medical University of Vienna Vienna Austria; ^5^ Ludwig Boltzmann Institute Applied Diagnostics Vienna Austria; ^6^ Molecular Radiopharmacy, INRaSTES, NCSR “Demokritos” Athens Greece

**Keywords:** bioisosteres, CuAAC, peptide‐based radiotracer, proteolytic stability, somatostatin, triazolo‐peptidomimetic

## Abstract

Radiolabeled peptides play a key role in nuclear medicine to selectively deliver radionuclides to malignancies for diagnosis (imaging) and therapy. Yet, their efficiency is often compromised by low metabolic stability. The use of 1,4‐disubstituted 1,2,3‐triazoles (1,4‐Tzs) as stable amide bond bioisosteres can increase the half‐life of peptides in vivo while maintaining their biological properties. Previously, the amide‐to‐triazole substitution strategy was used for the stabilization of the pansomatostatin radioligand [^111^In]In‐AT2S, resulting in the mono‐triazolo‐peptidomimetic [^111^In]In‐XG1, a radiotracer with moderately enhanced stability in vivo and retained ability to bind multiple somatostatin receptor (SSTR) subtypes. However, inclusion of additional 1,4‐Tz led to a loss of affinity towards SST_2_R, the receptor overexpressed by most SSTR‐positive cancers. To enhance further the stability of [^111^In]In‐XG1, alternative modifications at the enzymatically labile position Thr^10^‐Phe^11^ were employed. Three novel 1,4,7,10‐tetraazacyclododecane‐1,4,7,10‐tetraacetic acid (DOTA)‐peptide conjugates were synthesized with a 1,4‐Tz (Asn^5^‐*Ψ*[Tz]‐Phe^6^) and either a β‐amino acid (β‐Phe^11^), reduced amide bond (Thr^10^‐*Ψ*[NH]‐Phe^11^), or N‐methylated amino acid (*N*‐Me‐Phe^11^). Two of the new peptidomimetics were more stable in blood plasma in vitro than [^111^In]In‐XG1. Yet none of them retained high affinity towards SST_2_R. We demonstrate for the first time the combination of the amide‐to‐triazole substitution strategy with alternative stabilization methods to improve the metabolic stability of tumor‐targeting peptides.

## Graphical abstract

The use of 1,4‐disubstituted 1,2,3‐triazoles as stable amide bond bioisosteres can increase the half‐life of peptides in vivo while maintaining their biological properties. To further enhance the stability of [^111^In]In‐XG1, alternative modifications at the enzymatically labile position Thr^10^‐Phe^11^ were employed. Two of the new peptidomimetics were more stable in blood plasma in vitro than [^111^In]In‐XG1.

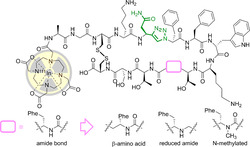

**Abbreviations**
1,4‐Tz: 1,4‐disubstituted 1,2,3‐triazole
1,4‐Tz, Tz: 1,4‐disubstituted 1,2,3‐triazole
^111^In: indium‐111
Aa: amino acid
ACN: acetonitrile
Bn: benzyl
Boc: tert‐butyloxycarbonyl
CuAAC: copper(I)‐catalyzed azide‐alkyne cycloaddition
DCM: dichloromethane
DIPEA: *N*,*N*‐diisopropylethylamine
DMSO: dimethyl sulfoxide
DMF: *N*,*N*‐dimethylformamide
DOTA: 1,4,7,10‐tetraazacyclododecane‐1,4,7,10‐tetraacetic acid
ESI: electrospray ionization
FA: formic acid
Fmoc: 9‐fluorenylmethyloxycarbonyl
GPCR: G‐protein coupled receptor
h: hour
HATU: hexafluorophosphate azabenzotriazole tetramethyl uronium
HPLC: high‐performance liquid chromatography
ISAˑHCl: imidazole‐1‐sulfonyl azide hydrochloride
MeOH: methanol
min: minutes
MTBE: methyl‐tert‐butyl ether
MS: mass spectrometry
MW: microwave
NEP: neprilysin
NET: neuroendocrine tumor
PET: positron emission tomography
RCP: radiochemical purity
RCY: radiochemical yield
RT: room temperature
r_t_
: retention time
SD: standard deviation
SPECT: single‐photon emission computed tomography
SPPS: solid‐phase peptide synthesis
SST‐14: somatostatin‐14
SSTR: somatostatin receptor
SI: supporting information
TATE: [Tyr^3^]octreotate
^t^Bu: tert‐butyl
TBTA: tris[(1‐benzyl‐1*H*‐1,2,3‐triazol‐4‐yl)methyl]amine
TFA: trifluoroacetic acid
TFE: trifluoroethanol
TIPS: triisopropylsilane
TOC: [Tyr^3^]octreotide
Trt: trityl

## 1 INTRODUCTION

Peptides are valuable biological vectors that are used in nuclear medicine for the delivery of radioactivity to tumors.^1^ When paired with diagnostic radiometals, used alongside the nuclear imaging modalities single‐photon emission computed tomography (SPECT) or positron emission tomography (PET), peptides can be used to evaluate the progression of a disease or for patient stratification.^2^ In addition, if peptides are labeled with therapeutic radiometals (α or β^−^ particle emitters), they can deliver a cytotoxic amount of radiation to unresectable tumors.^3^ Among the many virtues of these biomolecules, peptides stand out in nuclear medicine because of their capacity to interact with relevant oncological targets with high affinity, specificity and selectivity.^4^ Peptides also exhibit good tissue penetration and favorable pharmacokinetic properties that result in a rapid uptake of radioactivity in tumors and metastases as well as fast clearance from off‐target tissues. Yet, the proteolytic instability of peptides often prevents the clinical translation of peptide‐based radiotracers because of limited tumor uptake.^5^ Thus, several strategies aiming at the stabilization of peptides have been studied to address this issue.^6^ A promising approach makes use of metabolically stable amide bond bioisosteres.^7^ This way, peptides can be metabolically stabilized without altering their biological properties.

1,4‐Disubstituted 1,2,3‐triazoles (1,4‐Tz, Tz) have been extensively studied as metabolically stable *trans*‐amide bond mimetics.^8^ They share similar characteristics in terms of size, planarity, hydrogen bonding properties, and dipolar moment.^9^ However, this heterocyclic functional group cannot be hydrolyzed by peptidases. 1,4‐Tzs have been successfully employed in the metabolic stabilization of different radiolabeled peptides that are promising candidates for application in nuclear medicine (e.g., bombesin, neurotensin, minigastrin), leading to triazolo‐peptidomimetics with enhanced tumor uptake in mice bearing tumor xenografts in comparison to the respective all‐amide‐bond reference peptides.^10^, ^11^, ^12^ Yet, the exchange of *trans*‐amide bonds with a 1,4‐Tz can occasionally also result in a decrease of affinity of the peptidomimetics towards their respective receptor if the structural modification is not tolerated. In this regard, the insertion of a 1,4‐Tz increases the distance of neighboring amino acid side chains in the range of the effect of introducing β‐amino acids. In this situation, the combination of 1,4‐Tzs with alternative peptide stabilization strategies such as the use of other amide bond substituents and/or amino acids (D‐ or unnatural amino acids) within the same molecule could provide a solution.

**FIGURE 1:**
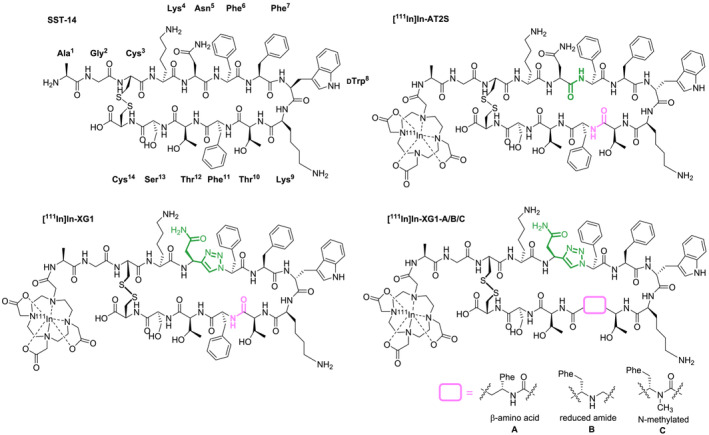
Structures of somatostatin‐14 (SST‐14) with numbered amino acids, [^111^In]In‐AT2S with enzymatically labile amide bonds depicted in green (Asn^5^‐Phe^6^) and pink (Thr^10^‐Phe^11^), [^111^In]In‐XG1 with the 1,4‐disubstituted 1,2,3‐triazole (1,4‐Tz) highlighted in green (Asn^5^‐*Ψ*[Tz]‐Phe^6^), and [^111^In]In‐XG1‐A/B/C, where the modification between Thr^10^ and Phe^11^ is represented by a pink box.

In a previous study, we used the amide‐to‐triazole substitution strategy for the first time to stabilize a radiotracer based on a cyclic peptide (Figure 1).^13^ The work focused on improving the in vivo stability of [^111^In]In‐AT2S, a radiotracer derived from the native somatostatin‐14 (SST‐14) with high binding affinity towards all somatostatin receptor (SSTR) subtypes (SST_1‐5_R).^14^ This feature makes [^111^In]In‐AT2S particularly interesting because it has the potential to address a wider range of cancer as compared with radiotracers that are mainly used in the management of SST_2_R‐overexpressing neuroendocrine tumors (NETs) (e.g., octreotide analogs 1,4,7,10‐tetraazacyclododecane‐1,4,7,10‐tetraacetic acid (DOTA)‐[Tyr^3^]octreotate (TATE), DOTA‐[Tyr^3^]octreotide (TOC)).^15^ Upon the introduction of a 1,4‐Tz in a position suspected to be prone to enzymatic cleavage (Asn^5^‐Phe^6^), only a moderate improvement in the in vivo stability was achieved while the affinity towards SST_1,2,3,5_R was maintained. In order to further stabilize the obtained triazolo‐peptidomimetic [^111^In]In‐XG1, other positions were explored for the potential introduction of an additional 1,4‐Tz moiety. Yet, the replacement of other amide bonds (Phe^6^‐Phe^7^, DTrp^8^‐Lys^9^, or Thr^10^‐Phe^11^) by 1,4‐Tz decreased significantly the affinity of the resulting peptidomimetics towards SST_2_R, the receptor subtype expressed by most cancers.^16^ We now set out to explore the utility of the combination of 1,4‐Tzs with other amide bond and amino acid substituents that are commonly used in the field of peptide sciences. Following this approach, three novel peptidomimetics of XG1 were synthesized. Each of them combined a 1,4‐Tz (Asn^5^‐*Ψ*[Tz]‐Phe^6^) with either a β‐amino acid (β‐Phe^11^), a reduced amide bond (Thr^10^‐*Ψ*[NH]‐Phe^11^), or a N‐methylated amino acid (*N*‐Me‐Phe^11^) at a position suspected to be enzymatically labile (Thr^10^‐Phe^11^), as depicted in Figure 1. To the best of our knowledge, this is the first report of the combination of the amide‐to‐triazole substitution strategy with alternative structural modifications to improve the metabolic stability of a radiolabeled tumor‐targeting peptide.

## 2 EXPERIMENTAL

### 2.1 Materials and methods

Reagents were purchased from Acros Organics (Geel, Belgium), Fluka (Buchs, Switzerland), Fluorochem (Hadfield, United Kingdom), Merck (Darmstadt, Germany), Novabiochem (Darmstadt, Germany), Sigma‐Aldrich (Buchs, Switzerland), and TCI (Zwijndrecht, Belgium) and were used without further purification. The preloaded resin H‐L‐Cys(Trt)‐2CT (0.71 mmol/g) and amino acids were bought from Iris Biotech (Marktredwitz, Germany), and DOTA‐tris(^t^Bu) ester was purchased from Chematech (Dijon, France). The radiolabeling was conducted using [^111^In]InCl_3_ from Curium (Petten, Netherlands). The loading of the 2CT resins was determined on an Agilent 8453 UV–visible spectroscopy system (λ = 301 nm, ε = 7800 L·mol^−1^·cm^−1^).^17^ The NanoDrop Micro‐UV/Vis Spectrophotometer at 280 nm (ε = 5625 L·mol^−1^·cm^−1^) was used to determine the concentration of the peptide aliquots. The coupling reactions that require microwave heating were conducted in the microwave‐assisted peptide synthesizer Biotage® Initiator+Alstra™. Reverse phase (RP)‐high‐performance liquid chromatography–mass spectrometry (HPLC‐MS) analyses of the peptides were conducted at 220 nm on an Agilent 1260 Infinity II system equipped with a Flexible pump, a 1260 VWD UV–Vis detector, and the LC/MSD system using the Acquity UPLC® Peptide BEH C18 column (300 Å, 1.7 μm, 2.1 mm × 100 mm). H_2_O (0.1% formic acid [FA]) and acetonitrile (ACN) (0.1% FA) were used as mobile phases. The chiral‐HPLC measurements of the α‐amino alkyne 9‐fluorenylmethyloxycarbonyl (Fmoc)‐Asn(Trt)‐CCH were performed on a Thermo Scientific Dionex Ultimate 3000 UHPLC system equipped with a diode array detector and using the Dr Maisch, ReproSil Chiral‐NR column (8 μm, 4.6 mm × 250 mm). Hexane and isopropanol were used as mobile phases. γ‐HPLC analyses of the radiopeptides were performed on an Agilent 1260 Infinity II equipped with a Gabi Nova Gina radiodetector (Elysia Raytest) and using the Chromolith® RP‐18 performance column (4.6 mm × 100 mm). The purification of the peptide conjugates was accomplished by preparative RP‐HPLC on an Agilent 1260 Infinity system equipped with the Xselect® Peptide CSH™ C18 OBD™ Prep column (130 Å, 5 μm, 19 mm × 150 mm) using H_2_O (0.1% trifluoroacetic acid (TFA)) and ACN (0.1% TFA) as mobile phases. Mass determination was accomplished with an HR‐MS Bruker maXis™ UHR electrospray ionization (ESI) time of flight spectrometer. NMR measurements were recorded on a Bruker FT‐NMR Avance III 500 MHz spectrometer at 500.10 (^1^H) and 125.75 (^13^C) MHz at 298 K in CDCl_3_.

The radiolabeling with [^111^In]InCl_3_ was conducted in low protein‐binding Eppendorf tubes. The neoBlock heater with inserts for 0.5 mL Eppendorf tubes was used to accomplish the reaction.

The AR42J cell line was purchased from the American Type Culture Collection (ATCC).

The human plasma used in the in vitro stability assays was purchased from Sigma‐Aldrich (H4522‐20ML).

### 2.2 Synthesis

The peptide conjugates XG1 and XG1‐A/B/C were synthesized at a scale of 0.030 mmol following general procedures 1–7 described in the Supporting Information (Chapter 2: Synthesis of peptidomimetics). The synthesis of the building blocks Fmoc‐Asn(Trt)‐CCH and Fmoc‐Thr(^t^Bu)‐COH was conducted as previously reported.^13^

#### 2.2.1 Synthesis of XG1

XG1 was synthesized using the commercial amino acids Fmoc‐Ala‐OH, Fmoc‐Cys(Trt)‐OH, Fmoc‐Gly‐OH, Fmoc‐Lys(Boc)‐OH, Fmoc‐Phe‐OH, Fmoc‐Ser(^*t*^Bu)‐OH, Fmoc‐Thr(^*t*^Bu)‐OH, Fmoc‐DTrp(Boc)‐OH, and DOTA‐tris(^*t*^Bu)‐ester. The α‐amino alkyne Fmoc‐Asn(Trt)‐CCH was synthesized for the incorporation of the 1,4‐Tz via the copper(I)‐catalyzed azide‐alkyne cycloaddition (CuAAC) reaction. The peptide was isolated in satisfying yield (35%) and high purity (>95%, Figure S1) as a white solid.

[M+2H^+^]^+2^, [M+3H^+^]^+3^, [M+4H^+^]^+4^ calculated for C_93_H_130_N_24_O_25_S_2_: 1024.9627, 683.6440, and 512.9850, respectively. Found: 1024.9629, 683.6449, and 512.9857.

#### 2.2.2 Synthesis of XG1‐A

XG1‐A was synthesized using the commercial amino acids Fmoc‐Ala‐OH, Fmoc‐Cys(Trt)‐OH, Fmoc‐Gly‐OH, Fmoc‐Lys(Boc)‐OH, Fmoc‐Phe‐OH, Fmoc‐β‐Phe‐OH, Fmoc‐Ser(^*t*^Bu)‐OH, Fmoc‐Thr(^*t*^Bu)‐OH, Fmoc‐DTrp(Boc)‐OH, and DOTA‐tris(^*t*^Bu)‐ester. The α‐amino alkyne Fmoc‐Asn(Trt)‐CCH was synthesized for the incorporation of the 1,4‐Tz via the CuAAC reaction. The peptide was isolated in satisfying yield (32%) and high purity (>95%, Figure S2) as a white solid.

[M+2H^+^]^+2^, [M+3H^+^]^+3^, [M+4H^+^]^+4^ calculated for C_93_H_130_N_24_O_25_S_2_: 1024.9627, 683.6440, and 512.9850, respectively. Found: 1024.9641, 683.6454, and 512.9858.

#### 2.2.3 Synthesis of XG1‐B

XG1‐B was synthesized using the commercial amino acids Fmoc‐Ala‐OH, Fmoc‐Cys(Trt)‐OH, Fmoc‐Gly‐OH, Fmoc‐Lys(Boc)‐OH, Fmoc‐Phe‐OH, Fmoc‐Ser(^*t*^Bu)‐OH, Fmoc‐Thr(^*t*^Bu)‐OH, Fmoc‐DTrp(Boc)‐OH, and DOTA‐tris(^*t*^Bu)‐ester. The amino aldehyde Fmoc‐Thr(^t^Bu)‐COH was synthesized for the reductive amination, while the α‐amino alkyne Fmoc‐Asn(Trt)‐CCH was used for the incorporation of the 1,4‐Tz via the CuAAC reaction. The peptide was isolated in satisfying yield (27%) and high purity (>95%, Figure S3) as a white solid.

[M+2H^+^]^+2^, [M+3H^+^]^+3^, [M+4H^+^]^+4^ calculated for C_93_H_132_N_24_O_24_S_2_: 1017.9804, 678.9869, and 509.4902, respectively. Found: 1017.9724, 678.9848, and 509.4907.

#### 2.2.4 Synthesis of XG1‐C

XG1‐C was synthesized using the commercial amino acids Fmoc‐Ala‐OH, Fmoc‐Cys(Trt)‐OH, Fmoc‐Gly‐OH, Fmoc‐Lys(Boc)‐OH, Fmoc‐Phe‐OH, Fmoc‐*N*‐Me‐Phe‐OH, Fmoc‐Ser(^*t*^Bu)‐OH, Fmoc‐Thr(^*t*^Bu)‐OH, Fmoc‐DTrp(Boc)‐OH, and DOTA‐tris(^*t*^Bu)‐ester. The α‐amino alkyne Fmoc‐Asn(Trt)‐CCH was synthesized for the incorporation of the 1,4‐Tz via the CuAAC reaction. The peptide was isolated in reasonable yield (14%) and high purity (>95%, Figure S4) as a white solid.

[M+2H^+^]^+2^, [M+3H^+^]^+3^, [M+4H^+^]^+4^ calculated for C_94_H_132_N_24_O_25_S_2_: 1031.9745, 688.3161, and 516.4870, respectively. Found: 1031.9723, 688.3179, and 516.4904.

### 2.3 Radiolabeling

Triazolo‐peptidomimetics (typically 1 mg) were dissolved in Milipore water/ACN (9:1) to a concentration of 1 μM. The accurate concentration of the peptide aliquots was determined using a Nanodrop Micro‐UV/Vis system at a wavelength of 280 nm (ε = 5625 L·mol^−1^·cm^−1^). The radiolabeling was conducted by mixing 50 μL of [^111^In]InCl_3_ (20–30 MBq) in 50 mM HCl, 5 μL of peptide solution (1 μM), and 10 μL of an aqueous NaOAc (0.3 M) solution. The final pH value (4.2–4.5) of the radiolabeling solution was determined with pH strips (Macherey Nagel^©^). The reaction was conducted by heating the mixture at 95°C for 10–15 min. The quality control of the radiolabeling was accomplished by γ‐HPLC using a Chromolith® RP‐18 performance column (2 μm, 4.6 mm × 100 mm) with a linear gradient from 90% A/10% B to 50% A/50% B in 15 min (A: 0.1% aqueous TFA and B: ACN) at a flow rate of 3 mL/min (see Figures S5–S8 for the γ‐HPLC chromatograms). The radiolabeling reactions were performed multiple times (*n* = 3–5), and the final radiochemical purity (RCP) and radiochemical yield (RCY) were determined by integration of signals corresponding to the desired indium‐111 (^111^In)‐labeled product, free [^111^In]InCl_3_, and any radioactive side product present in the gamma‐HPLC chromatograms.

### 2.4 In vitro characterization

#### 2.4.1 Cell binding and internalization studies

The cell binding and internalization assays were performed by incubating the ^111^In‐labeled peptide conjugates with SST_2_R‐overexpressing AR42J cells in six well plates as previously described (*n* = 2–3 in triplicates).^13^ The results of the cell binding and internalization assays are reported in Figure S9.

#### 2.4.2 Metabolic stability in human plasma

The stability assays were conducted by incubating the ^111^In‐labeled peptide conjugates in human plasma at 37°C for 24 h (*n* = 2). The detailed description of the procedure is described elsewhere.^13^

## 3 RESULTS AND DISCUSSION

### 3.1 Synthesis

**FIGURE 2:**
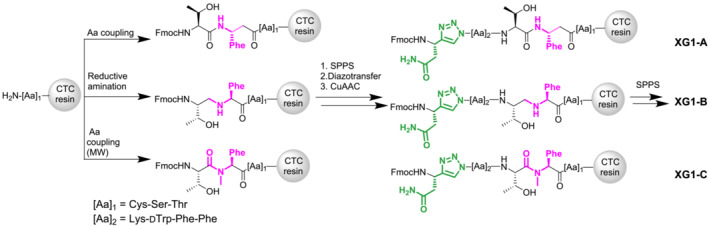
Introduction of different amino acid and amide bond substituents in position Thr^10^‐Phe^11^ and implementation of a 1,4‐disubstituted 1,2,3‐triazole (1,4‐Tz) in position Asn^5^‐Phe^6^ of XG1 on solid phase.

The synthesis of the triazolo‐peptidomimetics XG1 and XG1‐A/B/C was conducted as previously reported with small modifications (Figure 2).^13^ In brief, using a trityl resin pre‐loaded with the cysteine residue (Cys^14^), the amino acids were manually coupled following the conventional Fmoc/^t^Bu solid‐phase peptide synthesis (SPPS) protocol (Supporting Information, Chapter 2: Synthesis of peptidomimetics). Different strategies were followed for the implementation of the modification occurring at position Thr^10^‐Phe^11^. The incorporation of Fmoc‐β‐Phe‐OH (XG1‐A) into the peptide's backbone was achieved using hexafluorophosphate azabenzotriazole tetramethyl uronium (HATU) as a coupling reagent. In the case of XG1‐C, microwave heating was used to increase the yield of the coupling between the already incorporated *N*‐Me‐Phe^11^ and the following amino acid in the sequence, Fmoc‐Thr(^t^Bu)‐OH. As for the introduction of the reduced amide bond in XG1‐B, a different approach was taken combining both solid‐ and solution‐phase chemistry.^18^ First, Fmoc‐Thr(^*t*^Bu)‐OH was converted to the corresponding Weinreb amide followed by NaBH_3_CN reduction to its aldehyde. This aldehyde was then added to the resin with the anchored amino acid sequence (Phe‐Thr(^*t*^Bu)‐Ser(^*t*^Bu)‐Cys(Trt)) finished in the Fmoc‐deprotected N‐terminal phenylalanine (Phe^11^). The reduction of the newly formed imine was accomplished using an excess of NaBH_3_CN. The synthesis of the amino acid sequence of the triazolo‐peptidomimetics was accomplished in the same fashion for all the compounds. The anchored peptide was elongated till the position where the 1,4‐Tz was inserted (Asn^5^‐Phe^6^). First, the Fmoc‐protecting group of the N‐terminal amine of Phe^6^ was removed and converted into an azide using an excess of the diazo transfer reagent imidazole‐1‐sulfonyl azide hydrochloride (ISAˑHCl). The CuAAC reaction was accomplished by reaction of the azido‐functionalized peptide sequence on the resin with the α‐amino alkyne derived from Fmoc‐Asn(Trt)‐OH (Fmoc‐Asn(Trt)‐CCH) using [Cu(CH_3_CN)_4_]PF_6_ as catalyst and tris[(1‐benzyl‐1*H*‐1,2,3‐triazol‐4‐yl)methyl]amine (TBTA) as stabilizer.^19^ The sequence was continued by conventional Fmoc/^t^Bu SPPS chemistry and finished by appending the DOTA macrocyclic chelator at the N‐terminal amine (Ala^1^). The peptide was cleaved from the resin, and the trityl‐protecting groups were selectively removed. The disulfide bond‐driven cyclization was accomplished with an excess of iodine, resulting in full conversion to the cyclized peptide (>95%). The remaining protecting groups were removed with a cocktail containing TFA, and the cyclic peptidomimetics were purified by preparative HPLC. Due to challenging HPLC purification, a significant loss of the products was observed for the majority of peptidomimetics. This reduction in isolated yields was particularly significant for XG1‐C, the final yield of which did not exceed 15%. The synthesis of these peptide conjugates demonstrates that two different strategies aiming both at metabolic stabilization can be successfully combined for the preparation of a cyclic radiolabeled peptidomimetic using solid‐phase chemistry (Table 1). For complete HPLC‐MS characterization of the peptide conjugates, see Figures S1–S4.

**TABLE 1:** HPLC‐MS characterization of peptide conjugates.

Compound	Structure^a^	Purity^b^ [%]	*m/z*, [M+2H]^+2^ [Da]^c^ ^,^ ^d^
XG1	[DOTA,Asn^5^‐*Ψ*[Tz]‐Phe^6^,DTrp^8^]SST‐14	>95	1024.9629
XG1‐A	[DOTA,Asn^5^‐*Ψ*[Tz]‐Phe^6^,DTrp^8^,β‐Phe^11^]SST‐14	>95	1024.9641
XG1‐B	[DOTA,Asn^5^‐*Ψ*[Tz]‐Phe^6^,DTrp^8^,Thr^10^‐*Ψ*[NH]‐Phe^11^]SST‐14	>95	1017.9724
XG1‐C	[DOTA,Asn^5^‐*Ψ*[Tz]‐Phe^6^,DTrp^8^,*N*‐Me‐Phe^11^]SST‐14	>95	1031.9723

Abbreviations: DOTA, 1,4,7,10‐tetraazacyclododecane‐1,4,7,10‐tetraacetic acid; ESI, electrospray ionization; HPLC, high‐performance liquid chromatography; MS, mass spectrometry; SST‐14, somatostatin‐14.

^a^ *Ψ*[Tz] represents the *trans*‐amide bond replaced by a 1,4‐disubstituted 1,2,3‐triazole and *Ψ*[NH] a trans‐amide bond replaced by a reduced amide bond.

^b^ Purity was determined by RP‐HPLC.

^c^ Molecular masses of peptides were measured by ESI‐MS coupled to an HPLC system.

^d^ Calculated *m/z* of XG1 and XG1‐A = 1024.9627, XG1‐B = 1017.9804, and XG1‐C = 1031.9745.

### 3.2 Radiolabeling

The SPECT radiometal ^111^In was chosen for this study because of its widespread use in the oncological diagnosis of various tumors in nuclear medicine. ^111^In is often paired with SST_2_R‐avid peptidic tracers in the detection of NETs. The DOTA‐peptide conjugates were radiolabeled with [^111^In]InCl_3_ at molar activities of 5 MBq/nmol under standard radiolabeling conditions. RCY (determined by gamma‐HPLC) was >96% in all cases. High RCP values were achieved for XG1 (98.1 ± 0.7%), XG1‐A (98.0 ± 1.5%), and XG1‐B (98.1 ± 0.8%), while the ones for XG1‐C were found slightly lower (96.6 ± 1.4%). The high RCY and RCP (>96%) allowed the in vitro characterization of all the radiolabeled peptides without further purification. The characteristic γ‐chromatograms of each radiolabeled conjugate can be found in Figures S5–S8.

### 3.3 In vitro characterization

**FIGURE 3:**
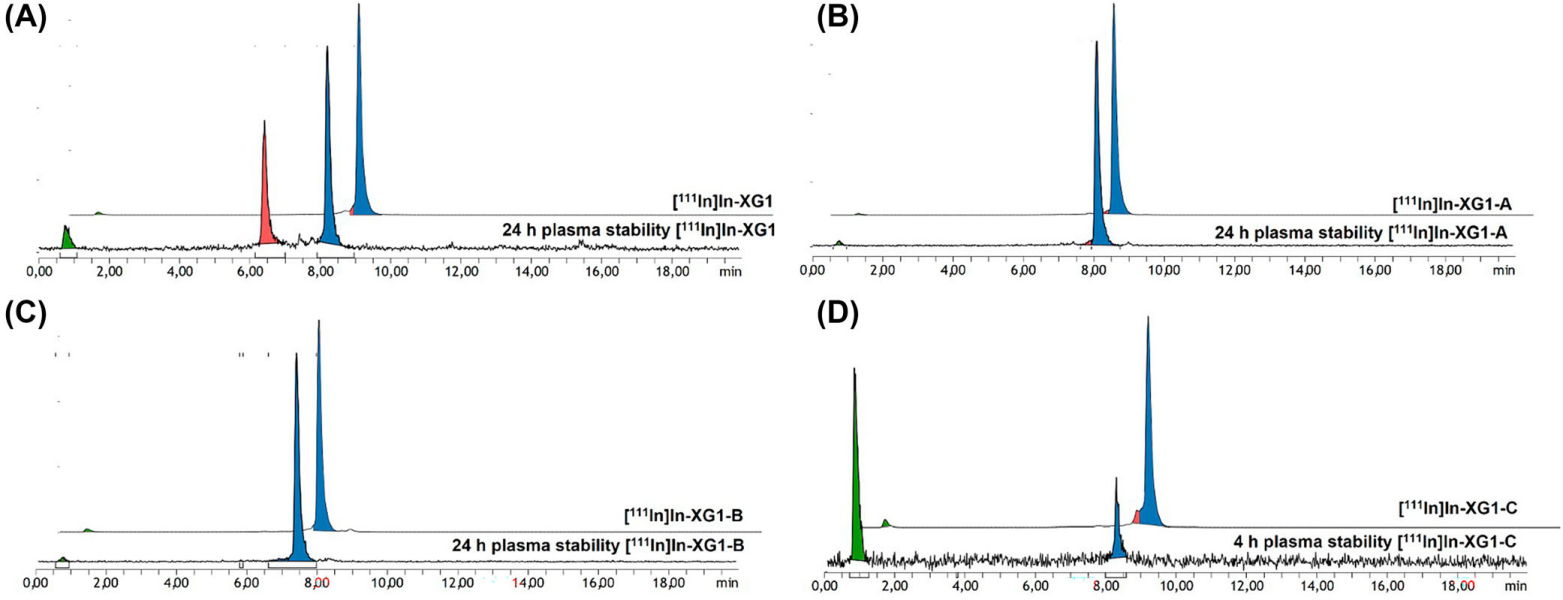
Comparison of γ‐high‐performance liquid chromatography (HPLC) chromatograms between the radiolabeled (A) [^111^In]In‐XG1, (B) [^111^In]In‐XG1‐A, (C) [^111^In]In‐XG1‐B, and (D) [^111^In]In‐XG1‐C and their respective metabolic profile 4 or 24 h after incubation in human plasma. Blue color depicts the intact radiolabeled peptide, whereas the radiometabolites are represented with either the green or red color.

In order to assess the proteolytic stability of the new radiolabeled peptide conjugates, in vitro stability studies were conducted in blood plasma. [^111^In]In‐XG1 and [^111^In]In‐XG1‐A/B/C were incubated in human plasma at 37°C, and samples were taken after 24 h for their analysis by γ‐HPLC (Figure 3). A total of 56% of intact [^111^In]In‐XG1 was found in plasma (r_t_ = 8.2 min), and a major radiometabolite was observed at r_t_ = 6.4 min (35.3%, Figure 3A). This result is in agreement with previously published data.^13^ The measurement of [^111^In]In‐XG1‐A and [^111^In]In‐XG1‐B revealed that the inclusion of either a β‐amino acid or a reduced amide bond enhanced the stability of the radioconjugates because more than 97% of intact peptide was found in both cases (Figure 3B,C). Unlike in the case of XG1, the formation of radiometabolites was successfully suppressed in the case of its derivatives XG1‐A and XG1‐B. These results suggest that Thr^10^‐Phe^11^ is indeed a labile amide bond implicated in the formation of the radiometabolite observed for XG1. This hypothesis is also supported by previously reported data that identified Thr^10^‐Phe^11^ as one of the main cleavage sites of the native SST‐14.^15^ Interestingly, [^111^In]In‐XG1‐C (r_t_ = 8.2 min) was completely degraded after 24 h, and only hydrophilic (likely low molecular weight) radiometabolites were observed (r_t_ = 0.8 min). To better understand the degradation of [^111^In]In‐XG1‐C over time, the stability was also determined after 4 h incubation in plasma (Figure 3D), after which only 27% of the original radiopeptide remained intact. Based on the observed radiometabolites, the break‐down of [^111^In]In‐XG1‐C differs significantly from that of [^111^In]In‐XG1. We hypothesize that the incorporation of the methylated amino acid (*N*‐Me‐Phe^11^) leads to a conformation of the peptide that facilitates the accessibility of amide bonds to proteases. Similar observations have been reported for other triazolo‐peptidomimetics.^11^ Even if plasma studies are useful to have an approximate estimation of the stability of a peptidic radiotracer, the absence of key enzymes in plasma might lead to results that do not represent the actual in vivo stability.^20^ For example, one of the main enzymes involved in the degradation of the native SST‐14 is neprilysin (NEP), a membrane‐bound metalloprotease abundantly expressed in various tissues.^21^, ^22^ The role of this enzyme in the degradation of peptides cannot be contemplated with in vitro plasma stability assays but would have to be assessed in vivo (e.g., mice).^23^

The binding of radiolabeled somatostatin derivatives to multiple SSTRs is of high interest because it allows to address a wide range of cancers with distinctive SSTR‐subtype expression patterns.^24^ Yet, SST_2_R is of major medical importance because it is over‐expressed or co‐expressed by most cancer types (e.g., NETs).^25^ Thus, we first focused on SST_2_R by conducting cell binding and internalization experiments with the SST_2_R‐expressing cell line AR42J. In order to determine the specificity of the binding and internalization of the radiolabeled peptides, the octapeptide TATE (DPhe^1^‐cycle(Cys^2^‐Phe^3^‐DTrp^4^‐Lys^5^‐Thr^6^‐Cys^7^)Thr^8^) was used in excess for blocking experiments. The measurement of [^111^In]In‐XG1 revealed that 24% of the total applied activity was bound to the cell membrane, of which 88% was internalized after 2 h. The blocking experiments with [^111^In]In‐XG1 in the presence of an excess of TATE showed <1% of unspecific binding, therefore confirming SST_2_R‐specific uptake. These values are in accordance with previously reported data.^13^ The new peptidomimetics [^111^In]In‐XG1‐A/B/C were subjected to the same experiment, but none of them displayed binding to or internalization into SST_2_R‐positive AR42J cells. From the total applied activity, <1% was found at the cell membrane and internalized, which corresponds to the determined unspecific binding (blocking experiments). See Figure S9 for the results of the binding and internalization studies. It is likely that the modifications at position Thr^10^‐Phe^11^ in the vicinity of the pharmacophore of the peptide (Phe^7^‐DTrp^8^‐Lys^9^‐Thr^10^) cause conformational changes preventing adequate fitting of the peptide ligand in the binding site of the receptor.^26^ Even though the new radiotracers could have the ability to bind the other SSTR subtypes, the inability to target tumors expressing the medically important SST_2_R disqualified them for radiotracer development. In order to diminish the risk of interfering with SST_2_R binding, other bonds than Thr^10^‐Phe^11^ could be subjected to modifications. For example, it has been demonstrated that modifications introduced in the neighboring position of an enzymatic cleavage site can also increase the peptide's metabolic stability.^12^ Thus, moving the modifications from Thr^10^‐Phe^11^ to the adjacent position, Phe^11^‐Thr^12^, holds potential for the stabilization of the peptidic radiotracers while maintaining their affinity towards SST_2_R and other SSTR subtypes. This work is currently ongoing and will be reported in due time.

## 4 CONCLUSION

We have explored for the first time the metabolic stabilization of a radiolabeled cyclic peptide by combining the amide‐to‐triazole substitution strategy with alternative amide bond/amino acid substitutions. According to in vitro stability studies, the combination of a 1,4‐Tz (Asn^5^‐*Ψ*[Tz]‐Phe^6^, XG1) with either a beta amino acid (β‐Phe^11^, XG1‐A) or a reduced amide bond (Thr^10^‐*Ψ*[NH]‐Phe^11^, XG1‐B) successfully stabilized the parent triazolo‐peptidomimetic XG1 in vitro. On the other hand, insertion of the N‐methylated amino acid *N*‐Me‐Phe^11^ (XG1‐C) at the same position was found to be detrimental to the stability of the peptidomimetics towards proteolytic degradation. Unexpectedly, all modifications at position Thr^10^‐Phe^11^ of XG1 in addition to the 1,4‐Tz compromised the binding of the peptide derivatives to SST_2_R. This suggests that the modification of other positions such as Phe^11^‐Thr^12^ should be investigated instead. We demonstrate that the combination of different approaches for the metabolic stabilization of medicinally interesting peptides is feasible, compatible with standard SPPS, and potentially applicable to other radiolabeled tumor‐targeting peptides. In addition, this research provides valuable information for future studies aiming at the stabilization of SST‐14‐based radiolabeled peptidomimetics.

## Supporting information


**Figure S1.** Structure and HPLC‐MS characterization of [^111^In]In‐XG1.
**Figure S2**. Structure and HPLC‐MS characterization of [^111^In]In‐XG1‐A.
**Figure S3**. Structure and HPLC‐MS characterization of [^111^In]In‐XG1‐B.
**Figure S4**. Structure and HPLC‐MS characterization of [^111^In]In‐XG1‐C.
**Figure S5**. γ‐HPLC of [^111^In]In‐XG1, r_t_= 8.20 min.
**Figure S6**. γ‐HPLC of [^111^In]In‐XG1‐A, r_t_= 8.07 min.
**Figure S7**. γ‐HPLC of [^111^In]In‐XG1‐B, r_t_= 7.37 min.
**Figure S8**. γ‐HPLC of [^111^In]In‐XG1‐C, r_t_= 8.25 min.
**Figure S9**. Cell binding and internalization of radiolabeled somatostatin derivatives [^111^In]In‐XG1 and [^111^In]In‐XG1‐A/B/C after 2 h using SST_2_R‐receptor expressing AR42J cells.
