# Supplementary material for: Combination of the amide‐to‐triazole substitution strategy with alternative structural modifications for the metabolic stabilization of tumor‐targeting, radiolabeled peptides
Source: J Pept Sci. 2024 Sep 11;31(1):e3654. doi: 10.1002/psc.3654 (PMC11602245; doi:10.1002/psc.3654)
Supplement: Supplementary file 1 — Figure S1. Structure and HPLC‐MS characterization of [111In]In‐XG1. Figure S2. Structure and HPLC‐MS characterization of [111In]In‐XG1‐A. Figure S3. Structure and HPLC‐MS characterization of [111In]In‐XG1‐B. Figure S4. Structure and HPLC‐MS characterization of [111In]In‐XG1‐C. Figure S5. γ‐HPLC of [111In]In‐XG1, rt= 8.20 min. Figure S6. γ‐HPLC of [111In]In‐XG1‐A, rt= 8.07 min. Figure S7. γ‐HPLC of [111In]In‐XG1‐B, rt= 7.37 min. Figure S8. γ‐HPLC of [111In]In‐XG1‐C, rt= 8.25 min. Figure S9. Cell binding and internalization of radiolabeled somatostatin derivatives [111In]In‐XG1 and [111In]In‐XG1‐A/B/C after 2 h using SST2R‐receptor expressing AR42J cells. [file PSC-31-e3654-s001.docx]

**Combination of the amide-to-triazole substitution strategy with alternative structural modifications for the metabolic stabilization of tumor-targeting, radiolabeled peptides**

**Xabier Guarrochena,^1,2,4^ Maximilian Anderla,^1,2,4,5^ Philipp Salomon^1,5^ Irene V. J. Feiner,^1,3,4^ Berthold A. Nock,^6^ Theodosia Maina,^6^ and Thomas L. Mindt^1,4,5*^**

^1^Institute of Inorganic Chemistry, Faculty of Chemistry, University of Vienna, Josef-Holaubek-Platz 2, 1090 Vienna, Austria; ^2^Vienna Doctoral School in Chemistry, University of Vienna, Währinger Straße 42, 1090 Vienna, Austria; ^3^Ludwig Boltzmann Institute Applied Diagnostics, AKH Wien c/o Sekretariat Nuklearmedizin, Währinger Gürtel 18-20, 1090 Vienna, Austria ^4^Department of Biomedical Imaging and Image Guided Therapy, Division of Nuclear Medicine, Medical University of Vienna, Währinger Gürtel 18-20, 1090 Vienna, Austria; ^5^Joint Applied Medicinal Radiochemistry Facility, University of Vienna and Medical University of Vienna, 1090 Vienna, Austria; ^6^Molecular Radiopharmacy, INRaSTES, NCSR “Demokritos”, 15341 Athens, Greece

*corresponding author: Thomas L. Mindt, e-mail: thomas [mindt@univie.ac.at](mailto:mindt@univie.ac.at)

**1. TABLE OF CONTENTS**

1. TABLE OF CONTENTS 1
2. SYNTHESIS OF PEPTIDOMIMETICS 2
3. HPLC-MS CHARACTERIZATION OF PEPTIDOMIMETICS 4
4. γ-HPLC CHROMATOGRAMS 8
5. RESULTS OF CELL BINDING AND INTERNALIZATION STUDIES 9

**2. SYNTHESIS OF PEPTIDOMIMETICS**

**General procedure 1. Manual solid-phase peptide synthesis**

Cys(Trt)-preloaded-2-chlorotrityl resin (42 mg, 0.71 mmol/g) was added to a syringe fitted with a polypropylene frit and a teflon tap (Biotage^©^). The resin was swollen by adding DMF (3 x 3 mL). The coupling reactions were conducted by adding a mixture of HATU (0.06 mmol, 2 equiv.), DIPEA (0.15 mmol, 5 equiv.) and the corresponding amino acid (0.06 mmol, 2 equiv.) in DMF (3 mL). The suspension was shaken for 2 h at RT, the solvent was filtered off and the resin was washed with DCM (3 x3 mL) and DMF (3 x 3 mL). Slightly different conditions were used for the coupling of the DOTA chelator, where a DMF solution (3 mL) of DOTA-tris(^t^Bu)ester (0.09 mmol, 3 equiv.), HATU (0.135 mmol, 4.5 equiv.) and DIPEA (0.24 mmol, 8 equiv.) was added to the resin to then shake it for 2 h. This reaction was typically repeated to achieve full conversion.

**General procedure 2. Fmoc deprotection on resin**

The Fmoc-deprotection was accomplished by adding a 20% piperidine solution in DMF (3 x 3 mL) to the resin containing the peptide with the Fmoc-protected amino functional group at the end of the sequence. Following filtration, the resin was washed with DCM (3 x 3 mL) and DMF (3 x 3 mL). The loading of the first amino acid was calculated based on the UV titration (λ= 301 nm) of the fluorenylmethylpiperidine (ε=7800 mol^-1^dm^3^cm^-1^).

**General procedure 3. Coupling under MW conditions**

After following general procedure 1 for the coupling of Fmoc-*N*(Me)Phe-OH in the synthesis of XG1-C, special conditions were required for the coupling of the subsequent amino acid Fmoc-Thr(^t^Bu)-OH. The Fmoc deprotection was conducted according to general procedure 2. After deprotection a solution of Fmoc-Thr(^t^Bu)-OH (0.06 mmol, 2 equiv.), HATU (0.06 mmol, 2 equiv.) and DIPEA (0.15 mmol, 5 equiv.) in 3 mL of DMF was added to the resin and the syringe was placed in the MW reactor of a peptide synthesizer (Biotage® Initiator+Alstra^TM^). The suspension was stirred for 15 min under microwave heating (70 ºC) after which the solvent was filtered off. The reaction was repeated to allow improved conversion (typically around 70%) to the desired peptide sequence.

**General procedure 4. On resin azido functionalization of peptide N-terminus.**

To the resin containing the sequence with the N-terminal amine at the end, a solution of ISA·HCl (0.15 mmol, 5 equiv.), and DIPEA (0.18 mmol, 6 equiv.) in DMF (3 mL) was added. The suspension was shaken for 2 h at RT, the solvent was filtered off and the resin was washed with DCM (3 x 3 mL) and DMF (3 x 3 mL). The Punna-Finn colorimetric test was used to determine the presence of azide functional groups. In case of incomplete conversion, the reaction was repeated.

**General procedure 5. On resin copper(I) catalyzed alkyne azide cycloaddition (CuAAC)**

A solution of the α-amino alkyne Fmoc-Asn(Trt)-CCH (Building block **1**, 0.06 mmol, 2 equiv.), DIPEA (0.03 mmol, 1 equiv.), [Cu(CH_3_CN)_4_]PF_6_ (0.015 mmol, 0.5 equiv.) and TBTA (0.015 mmol, 0.5 equiv.) in DMF (3 mL) was added over the resin containing the sequence with the azide functional group at the N-terminus. The reaction was shaken over night at RT. The content of the syringe was filtered off and the resin was washed with a 0.5% sodium diethyldithiocarbamate solution in DMF (3-5 x 3 mL) followed by DMC (3 x 3 mL) and DMF (3 x 3mL). To verify the presence of unreacted azide groups the Punna-Finn test was performed as well as HPLC-MS analyses. ^27^. The reaction was repeated in case of incomplete conversion.

**General procedure 6. On resin reductive amination**

Following the removal of the Fmoc protecting group with 20% piperidine solution (3 x 3 mL), the resin was washed with 3 mL of each DMF, MeOH and DCM. Next, the α-amino aldehyde Fmoc-Thr(^t^Bu)-CHO (Building block **2**, 0.45 mmol, 15 equiv.) was added to the resin in a 1M solution in DMF. The suspension was shaken for 10 min and NaBH_3_CN (0.45 mmol, 15 equiv.) was added in a 1M solution of DCM/MeOH 3:1 + 1% AcOH. The mixture was shaken for an additional hour, the solvent was removed by filtration and the resin was washed with 3 mL of each DMF, MeOH and DCM. Then, the resin was treated with a solution of di-*tert*-butyl dicarbonate (0.090 mmol, 3 equiv.) and DIPEA (0.090 mmol, 3 equiv.) in DMF (3 mL) and shaken overnight. The resin was washed with 3 mL of each DMF, MeOH and DCM and the completion of the reductive amination was confirmed by HPLC-MS. The reaction was repeated in case of incomplete conversion.

**General procedure 7. Peptide cleavage, cyclization, complete deprotection and purification**

To cleave the entire sequence from the resin and selectively remove the trityl (Trt) protecting groups of the cysteine residues, a cleavage cocktail containing DCM/TFE/AcOH (7/2/1, 3 mL) was added to the syringe. After shaking for 2 h at RT, the resin was filtered, and the solution was added over a 0.8 M solution of iodine (0.3 mmol, 10 equiv.) in a DCM/TFE/AcOH (7:2:1) mixture. The reaction was let stir at RT and conversion to the cyclized peptide was determined by HPLC-MS (typically 30 min). Following completion, the iodine was quenched with a 1 M aqueous solution of ascorbic acid (2 mL) and the mixture was extracted with DCM (3 x 3 mL). The organic layers were combined and washed with a 5% aqueous solution of NaCl/Citric acid (1:1, 3 x 3 mL). The organic phase was concentrated *in vacuo* and the full deprotection of the side-chains of the amino acids was achieved by adding 4 mL of a cocktail containing TFA/TIPS/H_2_O (9.5:0.25:0.25). The reaction was stirred for 4 h at RT and the resulting solution was washed with heptane (3 x 2 mL) and concentrated with a stream of argon. 3 mL of ice-cold MTBE were added to precipitate the peptide and the mixture was vortexed, sonicated and centrifuged (4000 rpm, 5 min) finally discarding the supernatant. This process was repeated 3 times. The preparative-HPLC purification was accomplished by dissolving the crude peptide (approximately 10 mg) in 1 mL of a A/B (80:20) mixture containing 0.1% TFA and were purified by preparative HPLC eluted at a flow rate of 17 mL/min with the linear gradient from 80% A/20% B to 40% A/60% B in 30 min (A: 0.1% aqueous TFA and B: ACN).

**3. HPLC-MS CHARACTERIZATION OF PEPTIDOMIMETICS**

**XG1**

**
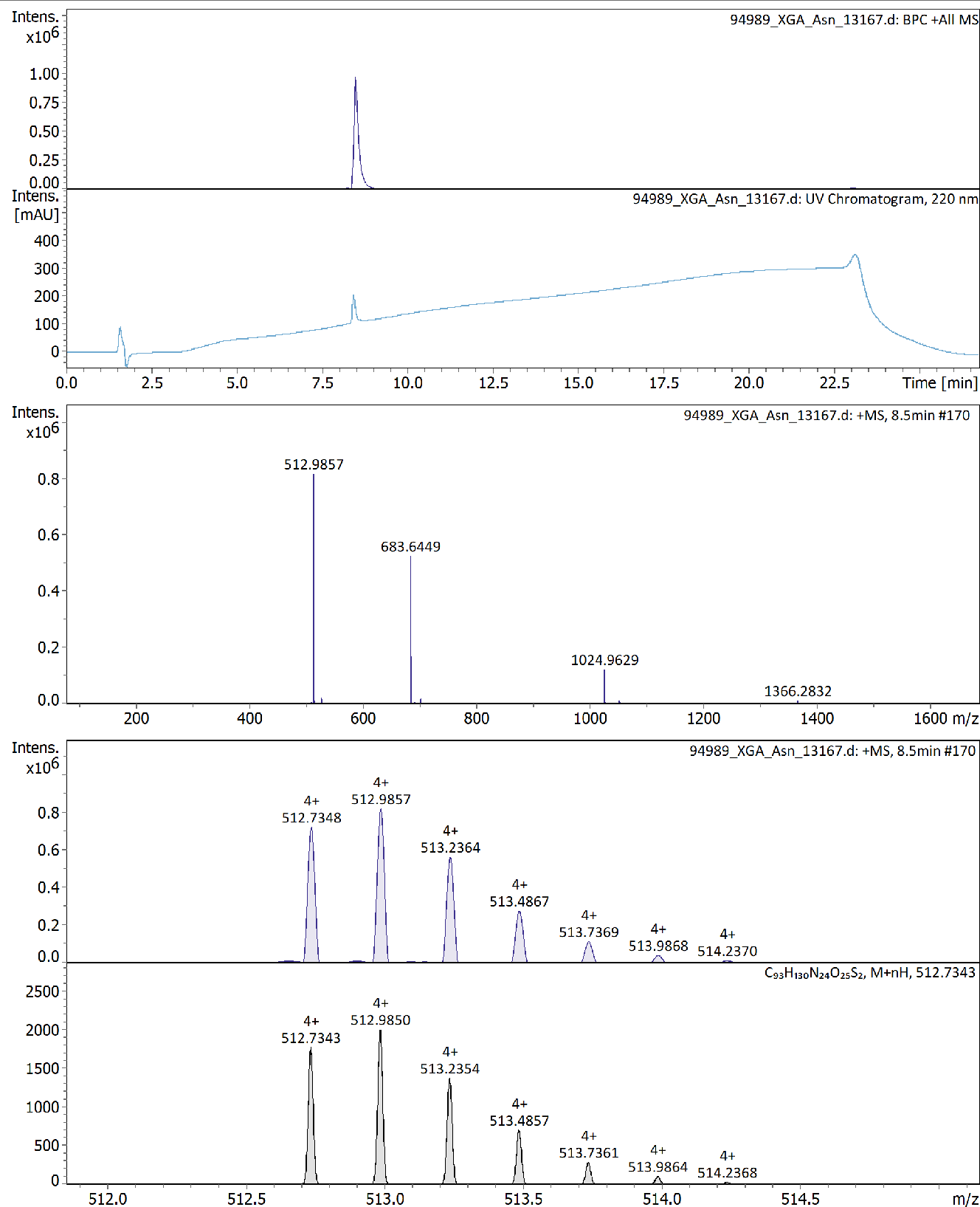
**

**Figure S1: Structure and HPLC-MS characterization of [^111^In]In-XG1**

**XG1-A**

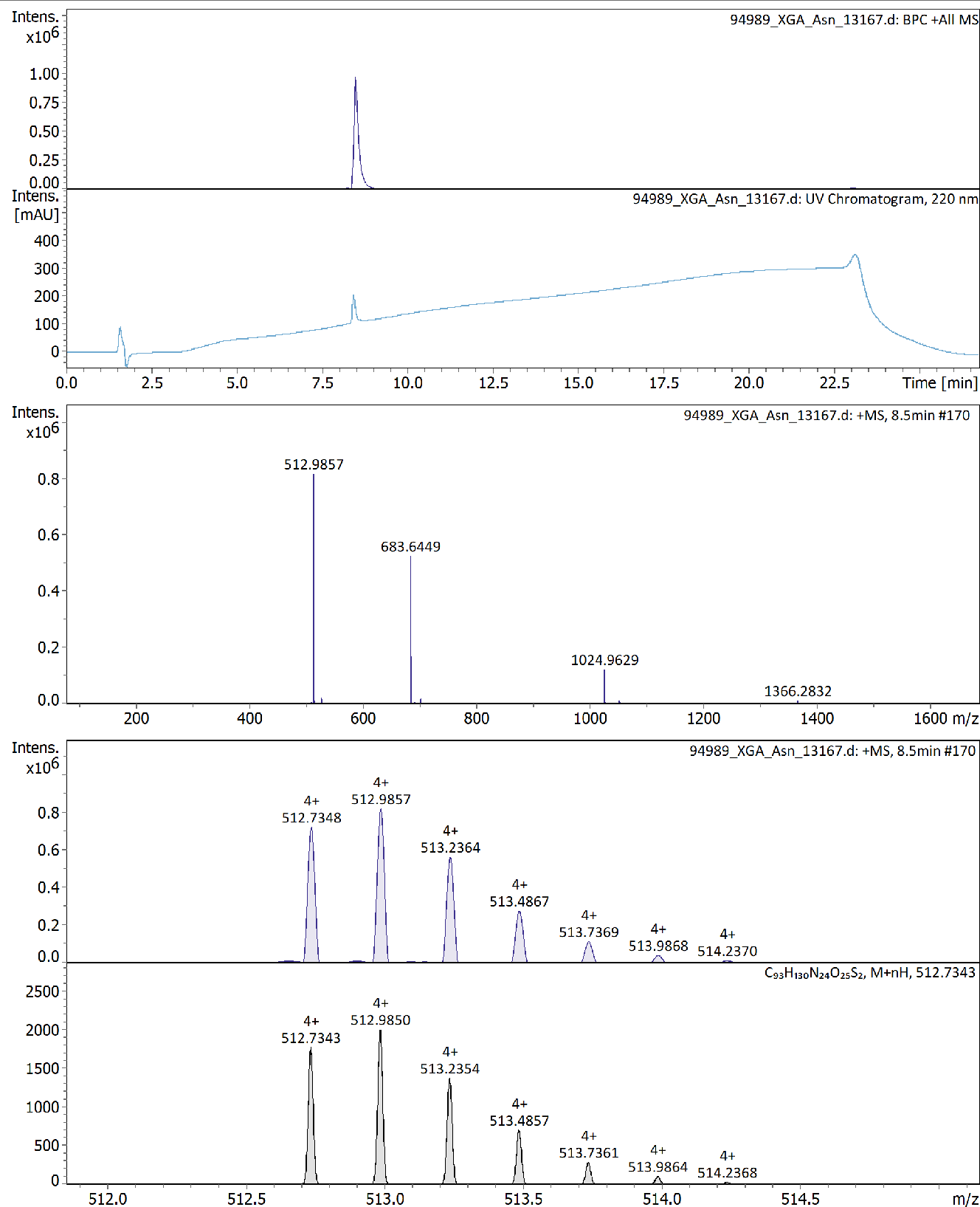


**Figure S2: Structure and HPLC-MS characterization of [^111^In]In-XG1-A**

**XG1-B**

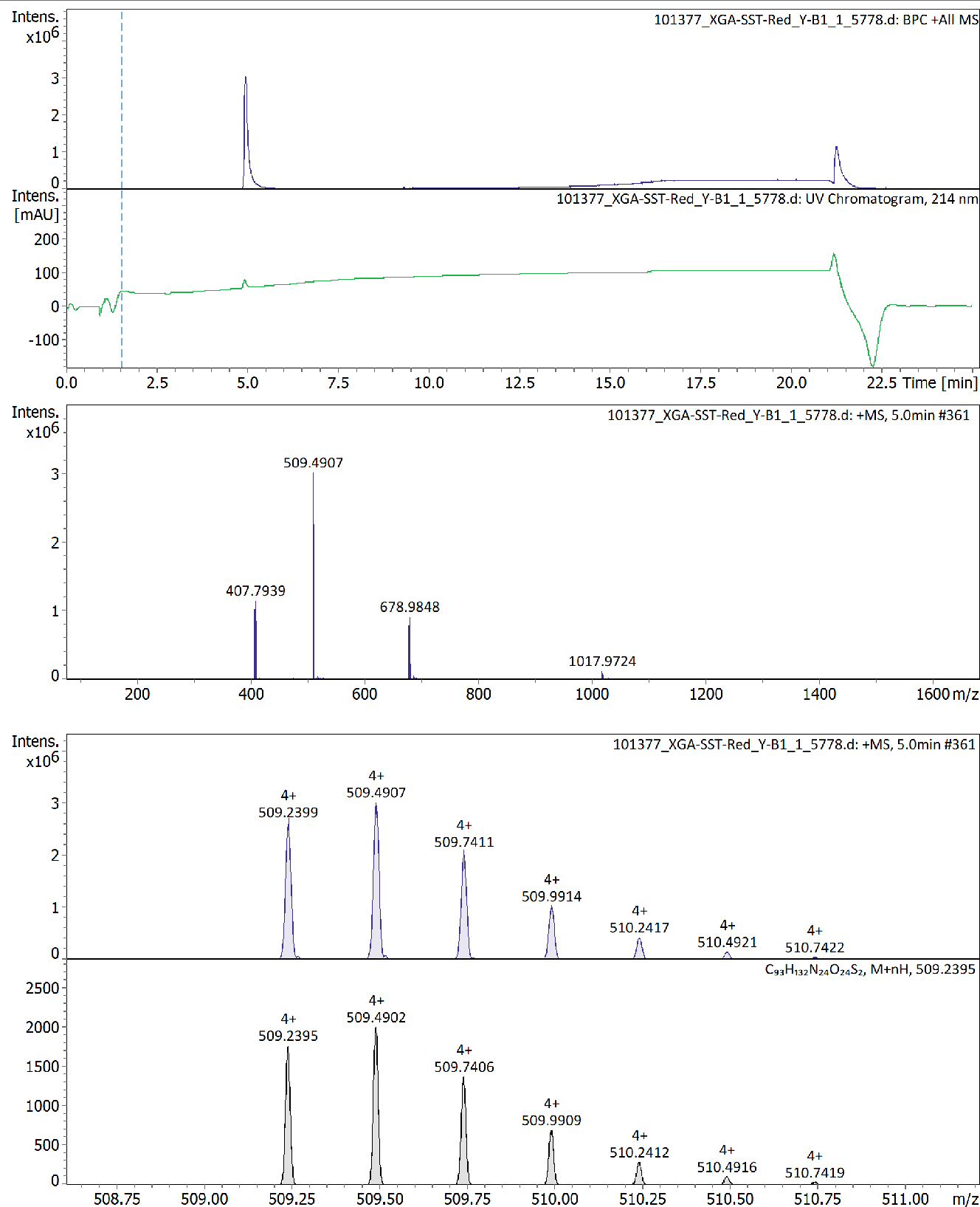


**Figure S3: Structure and HPLC-MS characterization of [^111^In]In-XG1-B**

**XG1-C**

**
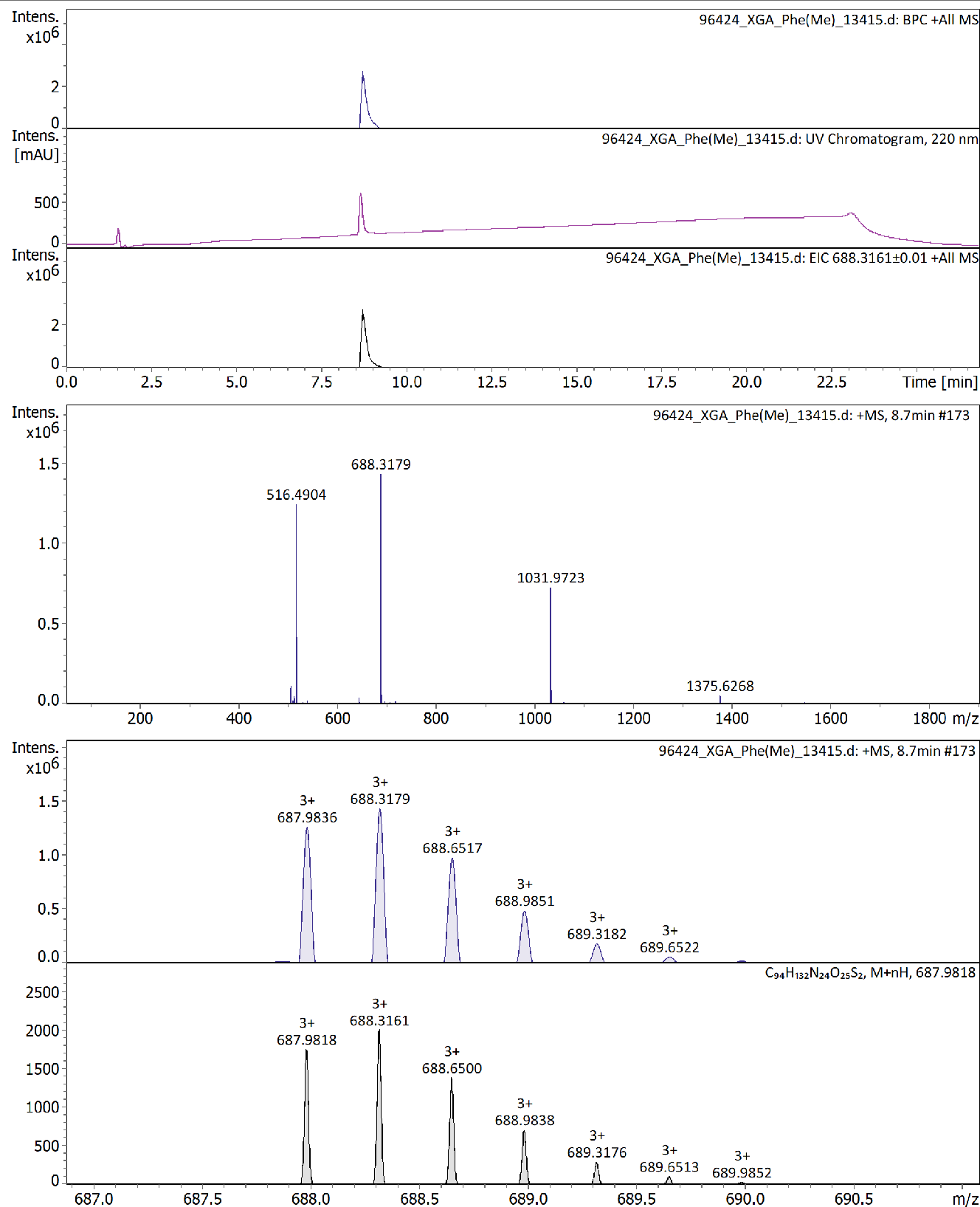
**

**Figure S4: Structure and HPLC-MS characterization of [^111^In]In-XG1-C**

**4. γ-HPLC CHROMATOGRAMS**

**[^111^In]In-XG1**

**
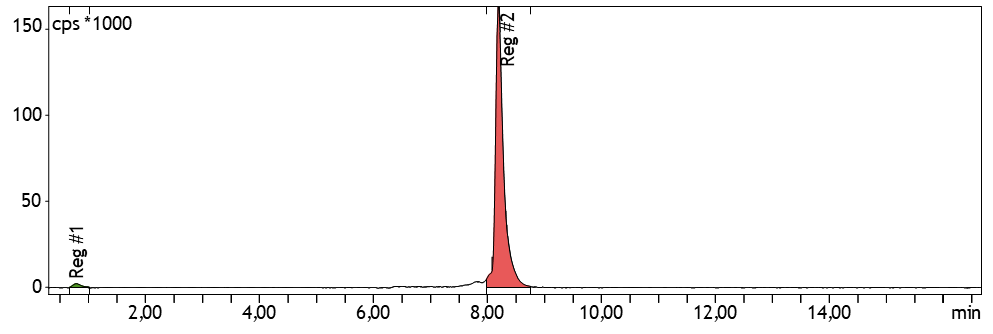
**

**Figure S5: γ-HPLC of [^111^In]In-XG1, r_t_= 8.20 min**

**[^111^In]In-XG1-A**

**
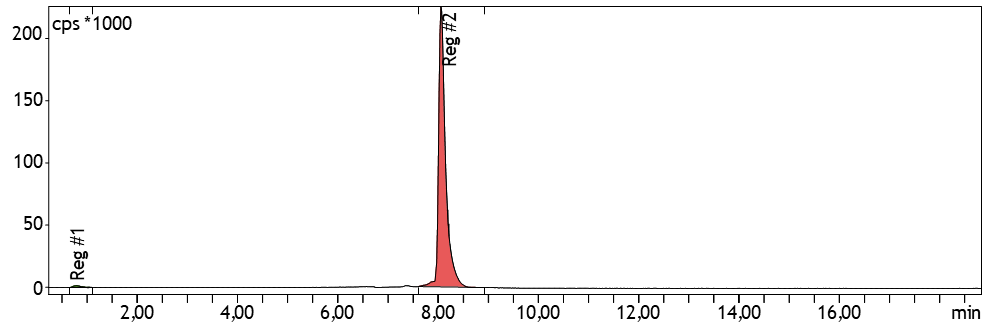
**

**Figure S6: γ-HPLC of [^111^In]In-XG1-A, r_t_= 8.07 min**

**[^111^In]In-XG1-B**

**
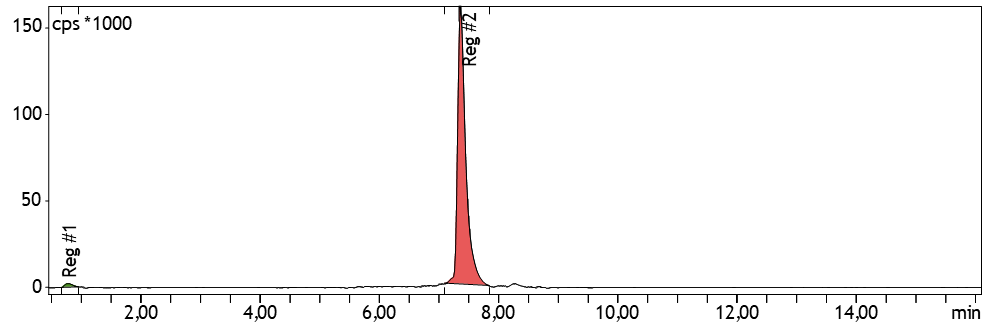
**

**Figure S7: γ-HPLC of [^111^In]In-XG1-B, r_t_= 7.37 min**

**[^111^In]In-XG1-C**

**
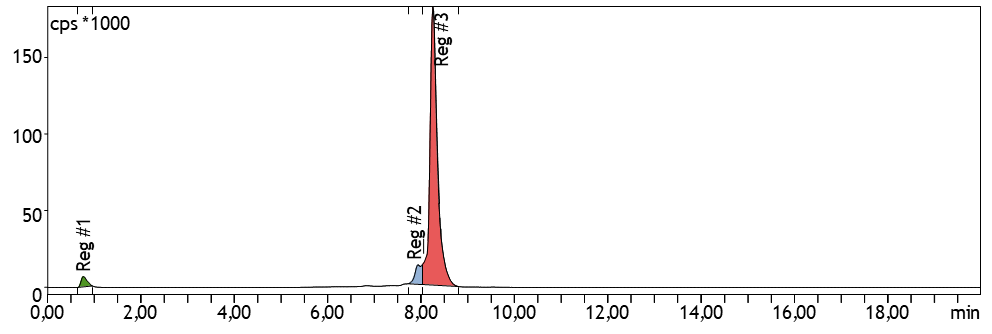
**

**Figure S8: γ-HPLC of [^111^In]In-XG1-C, r_t_= 8.25 min**

**5. RESULTS OF CELL BINDING AND INTERNALIZATION STUDIES**

**
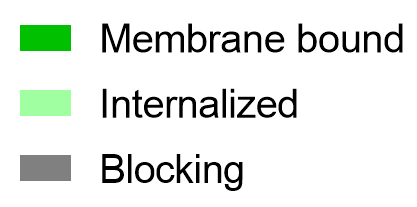
**

**
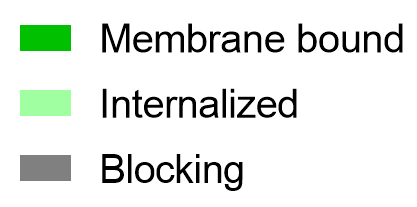
**

**Figure S9: Cell binding and internalization of radiolabeled somatostatin derivatives [^111^In]In-XG1 and [^111^In]In-XG1-A/B/C after 2 h using SST_2_R-receptor expressing AR42J cells.**
